# Structured matter creates toroidal structured light

**DOI:** 10.1038/s41377-022-00927-0

**Published:** 2022-07-22

**Authors:** Andrew Forbes

**Affiliations:** School of Physics, University of the Witwatersrand, Johannesburg, South Africa

**Keywords:** Optical techniques, Optical materials and structures

## Abstract

Nano-structured metasurfaces have to be tailored from artificial atoms that act as toroidal emitters, giving rise to a new form of light long predicted: “flying doughnuts” as propagating spatial-temporal electromagnetic toroidal pulses in both the visible and THz regimes.

A toroidal topology can be formed by a surface of revolution about a central point, for example, rotating a small radius disk about a large radius of revolution will result in a doughnut-shaped toroid with a hole in the middle, the name derived from the popular sugary pastry. If this is done mechanically, as a child might do to make a doughnut out of play-dough, then the matter toroid exists as a 3D object in space. But it has long been predicted that such topologies might exist in light itself^1^. For this to be realized means the control of light in 3D space and 1D time for 4D structured light^2^. Reporting recently^3^, Nikolay Zheludev and collaborators have demonstrated that nano-structured metasurfaces can be carefully crafted to exhibit the necessary resonances, acting as artificial toroidal emitters. The result is exotic forms of electromagnetic waves propagating in free space, from optical to THz frequencies.

In our common textbook description of light the electromagnetic disturbance is confined to the 2D plane transverse to the direction of propagation, with the electric and magnetic fields always orthogonal to one another. To create toroidal pulses of light, the electric and magnetic fields remain orthogonal, but now in loops: the electric field looping around the inner circumference of the doughnut and the magnetic field about the outer circumference, or vice versa. But how can one make such exotic states of light? The answer requires concepts from beyond the textbook examples of dipole emitters and towards toroidal emitters, but such higher-order and toroidal multipoles have been challenging to harness for new radiative states of light^4^.

An essential ingredient in such toroidal pulses is coupling between the spatial and temporal components of the field, so that it lives in a 4D space as a non-separable state, i.e., that the space and time components cannot be factored as a product of independent space-like and time-like terms. To do this, the team used a clever combination of structured matter excited by transversely structured light. The structured matter in the optical realization was a nano-structured metasurface comprising radial plasmonic rings, with narrower rings at the center of the metasurface and broader rings towards the edges. The varying width of the plasmonic rings leads to a gradual redshift of the transmission resonance along the radial direction profile, ensuring that space and time (through frequency/wavelength control) are coupled. To make this spatial wavelength sweep appear 1D, the team excited the metasurface with a radially polarized beam, so that each radial polarization vector sees only a 1D grating structure in the radial direction. The control needed in the pupil plane for true toroidal pulse creation is exacting, and only possible by state-of-the-art engineered metasurfaces. In the THz band, the input was a linear polarized beam, and the excitation of the metasurface atoms non-linear, in one step creating both the radially polarized structure and the wavelength sweep (Fig. 1).

**Fig. 1:**
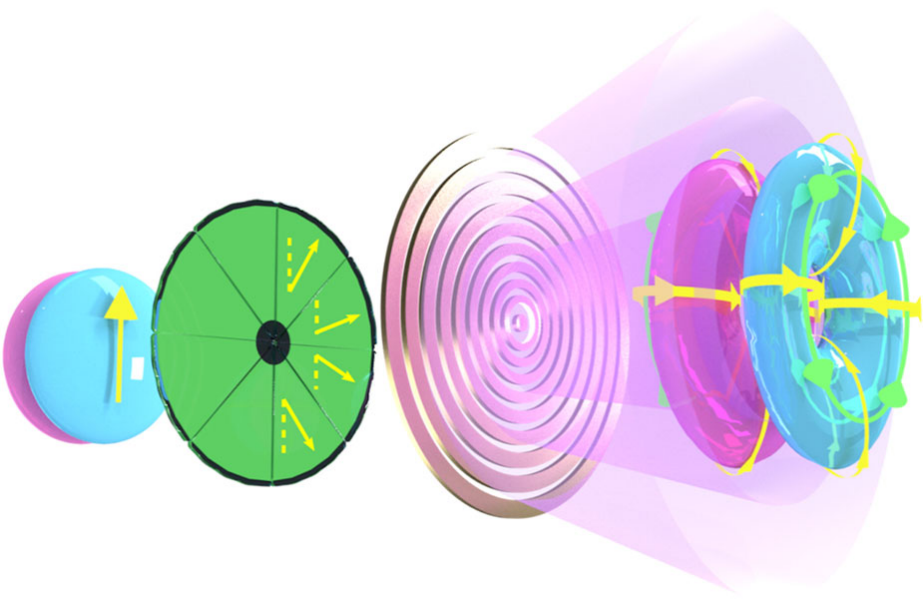
Crafting toroidal light. A conventional linear polarized field is first converted into a radially polarized beam, with the electric field pointing radially outwards, as depicted by the yellow arrows. This is passed through a metasurface comprising gold rings of varying width, narrower near the center and increasing in width towards the rim. The radially polarized light sees this as a 1D grating, with the resulting toroidal response leading to toroidal pulses of light: doughnut-shaped pulses with looping electric (yellow) and magnetic (green) fields. Image courtesy of the University of Southampton

A major challenge yet remained: how to characterize such pulses of light? The authors deploy a myriad of tools, from traditional interferometry and hyperspectral imaging to novel quantum-inspired decompositions^5^, which together could piece together the puzzle of this new form of light. The result is a demonstration of toroidal pulses with fidelities of about 80%, for a convincing first look.

The results by the team can be contextualized by comparing it to the simultaneous report^6^ of scalar toroidal pulses by the group of Qiwen Zhan. In the present work, the approach is to use toroidal emitters to create propagating electromagnetic vector toroidal pulses, requiring sophisticated nano-structured metasurfaces. In the competing work, the approach is to by-pass the need for the emitters and instead fold light conformally in on itself, from a tube to a toroid^7^. While the present work is vectorial and the former scalar (with amplitude and phase independent of the polarization), both require carefully designed optical elements and set-ups, and both used highly sophisticated characterization tools to get just a glimpse of the result.

Despite these exciting advances, there is still work to be done. Neither report has shown any application of the toroidal pulses, whereas it has been suggested that their non-separable space–time structure could make them invaluable for a wide range of applications, including as the basis for new forms of spectroscopy, as quantum qubits and for fundamental tests of new Aharonov–Bohm effects. These exciting opportunities are sure to follow.

Space–time coupling in ultrafast light is an ever-present phenomenon that happens naturally in light propagation and focussing, making initially separable states non-separable. The present work shows that this non-separability can be engineered through careful construction of the pupil plane function, structuring matter for structured light. That we can create new forms of light long predicted is a tremendous achievement, and an exciting prospect for the community at large.
